# Digital Management Systems in Academic Health Sciences Laboratories: A Scoping Review

**DOI:** 10.3390/healthcare9060739

**Published:** 2021-06-16

**Authors:** Margareth Timóteo, Emanuelle Lourenço, Ana Carolina Brochado, Luciana Domenico, Joice da Silva, Bruna Oliveira, Renata Barbosa, Pietro Montemezzi, Carlos Fernando de Almeida Barros Mourão, Beni Olej, Gutemberg Alves

**Affiliations:** 1 Clinical Research Unit, Antônio Pedro Hospital, Fluminense Federal University, Niteroi 24020-140, Brazil; margatimoteo@gmail.com (M.T.); ldqueiroz@id.uff.br (L.D.); joicepearl@gmail.com (J.d.S.); brunsoliver4@gmail.com (B.O.); beniolej@id.uff.br (B.O.); 2 Post-Graduation Program in Medical Sciences, Fluminense Federal University, Niteroi 24020-140, Brazil; 3 Post-Graduation Program in Dentistry, Fluminense Federal University, Niteroi 24020-140, Brazil; emanuelle_stellet@yahoo.com.br; 4 Post-Graduation Program in Science and Biotechnology, Fluminense Federal University, Niteroi 24020-140, Brazil; anacarol.batista.b@gmail.com (A.C.B.); renata_licaa@hotmail.com (R.B.); 5 Independent Researcher, 24128 Bergamo, Italy; m.montemezzi@libero.it

**Keywords:** scoping review, academic health centers, software, laboratory management

## Abstract

Good laboratory practices (GLP) increase the quality and traceability of results in health sciences research. However, factors such as high staff turnover, insufficient resources, and a lack of training for managers may limit their implementation in research and academic laboratories. This Scoping Review aimed to identify digital tools for managing academic health sciences and experimental medicine laboratories and their relationship with good practices. Following the PRISMA-ScR 2018 criteria, a search strategy was conducted until April 2021 in the databases PUBMED, Web of Sciences, and Health Virtual Library. A critical appraisal of the selected references was conducted, followed by data charting. The search identified twenty-one eligible articles, mainly originated from high-income countries, describing the development and/or implementation of thirty-two electronic management systems. Most studies described software functionalities, while nine evaluated and discussed impacts on management, reporting both improvements in the workflow and system limitations during implementation. In general, the studies point to a contribution to different management issues related to GLP principles. In conclusion, this review identified evolving evidence that digital laboratory management systems may represent important tools in compliance with the principles of good practices in experimental medicine and health sciences research.

## 1. Introduction

Laboratory research plays an essential role in providing evidence for translational medicine and sustainable solutions to healthcare [1]. However, the reliance on experimental medicine demands increased traceability and data integrity, ensuring the quality of transferrable results to the clinical setting. In recent years, the scientific community experienced awareness regarding a reproducibility crisis related to factors such as the pressure for publication, low statistical power, and insufficient supervision [2]. On the other hand, adequate management, training, and good practices may increase data quality by improving workflow, avoiding errors, and providing traceability [2].

Good laboratory practices (GLP) may be defined as a quality system encompassing organizational processes and conditions under which studies are planned, executed, monitored, registered, and reported [3]. The Principles of Good Laboratory Practice were first developed by a group of GLP experts led by the USA, established in 1978 under the Special Program on the Control of Chemicals, based on the FDA’s regulations for non-clinical laboratory studies. The Organization for Economic Cooperation and Development (OECD) published the Principles of Good Laboratory Practice and Compliance Monitoring in January 1998 [3]. Since then, it represents the primary set of standards available worldwide to ensure quality, reliability, and integrity, providing a solid approach to the management of research laboratories [4].

However, academic laboratories experience several critical barriers to developing and implementing a GLP-compliant infrastructure [5]. These limitations include poor training on management, lack of funding for compliance costs, and high staff turnover due to a dependence on students as temporary personnel [6]. Therefore, laboratory managers at academic centers should explore tools that facilitate supervision and identify critical steps in the laboratory workflow. In this context, digital systems are among the most important tools available for efficient management, ranging from dedicated computer programs to smartphone applications. Laboratory information management systems (LIMS) offer databases and automation [7] that allow experimental data tracking and storage [8]. Other software and digital services that fall outside of the original LIMS classification provide a broader offer of solutions to laboratory management [6,9], coping with other aspects of quality assurance related to communication, staff, multiuser equipment schedule and maintenance, standard procedures, and inventory control, which are fundamental in the full spectrum of a laboratory’s workflow [10,11].

Despite the potential effectiveness of these digital tools in meeting specific aspects of laboratory management, it remains unclear how these systems may directly or indirectly contribute to adherence to the GLP principles. In this context, the present review aimed to provide evidence on the theme by scoping the scientific literature for the available digital tools designed to manage health sciences and experimental medicine laboratories and discuss the assessments of effectiveness, acceptance, and their potential for compliance to different aspects of good laboratory practices.

## 2. Materials and Methods

### 2.1. Protocol and Registration

This review followed the PRISMA recommendations for scoping reviews (PRISMA-ScR) [12], as shown in the Supplementary Table S1. The study protocol was registered in the Open Science Framework database under the Digital Object Identifier doi:10.17605/OSF.IO/KPC3Q on 15 July 2020.

### 2.2. Sources of Information and Research Strategy

The broad question that guided the review was: “Are there available digital tools for the management of academic health sciences laboratories?” Strategies were developed to search for data sources in three different databases: PUBMED (www.ncbi.nlm.nih.gov/pubmed (accessed on 26 April 2021)), Web of Science (WoS) (clarivate.com/webofsciencegroup (accessed on 26 April 2021)), and the Virtual Health Library (VHL) (bvsalud.org (accessed on 26 April 2021)). The research was carried until April 2021. Grey literature was consulted through the OpenGrey Database (available at http://www.opengrey.eu/ (accessed on 24 May 2021)). The search keys are described in Table 1, with various combinations of Medical Subject Headings (MeSH) descriptors selected to cover as many articles as possible coping with management software approaches in academic or research settings.

### 2.3. Selection of Sources of Evidence

The eligibility criteria were determined on a PIO (Population, Intervention, Outcome) variant of the PICO framework for the selection of studies, more adequate for qualitative reviews [13].

A structured question was produced, in which, Population (P): academic health sciences laboratories, Intervention (I): the use of digital tools, and Outcomes (O): management for quality. After the references were retrieved from the database search, a group of five trained and calibrated reviewers read all titles and abstracts, applying the eligibility criteria, which included complete works on digital tools that aid in the administration of laboratories in academic or research environments, in health or biomedical sciences, including collections and biorepositories. Studies were excluded if they (i) were entirely out of the subject, (ii) did not address laboratory management, (iii) did not deal with software or digital tools, and (iv) were not proposed or discussed for health sciences or biomedical research. Additionally, articles on software that exclusively assessed experimental data management were considered outside the scope of this review. The inter-examiner reliability was assessed through simultaneous assessment of references by five evaluators, obtaining a Cohen’s Kappa coefficient of 0.93. Doubts and disagreements were resolved in weekly meetings conducted during this stage.

### 2.4. Critical Appraisal

A critical appraisal was conducted with the selected references, applying an instrument described by Whittemore and Knafl [14], considering two relevant criteria: (i) methodological and theoretical soundness and (ii) relevance of the data to the proposed question of the review. The methodological assessment considered whether studies presented adequate identification and traceability of the software, evaluating effectiveness, applicability, or acceptance. The adherence to the review’s question was considered according to the description of management functions, target users and environment, and software limitations. Each present parameter was scored with 1 point, to a maximum of 4 points. No study was excluded based on this assessment classification, even though the score was included as a variable in the data analysis stage. In general, studies of lower scores contributed less to the analytical process.

### 2.5. Synthesis of Results and Data Charting

The main characteristics of the selected studies were collected and tabulated, including year and country of conduction, name and type of digital tool, topics of laboratory management issued by the software, target public and environment of application, accessibility, and whether the software was free or paid. The data extraction was performed in conjunction with five authors in regular meetings. A specific table was produced solely with the studies that performed evaluations of effectiveness or acceptance, with the respective outcomes. A chart was produced connecting the management topics issued by the different tools and the respective sections/chapters from the Organization for Economic Cooperation and Development (OECD) GLP Principles [3].

## 3. Results

**Figure 1:**
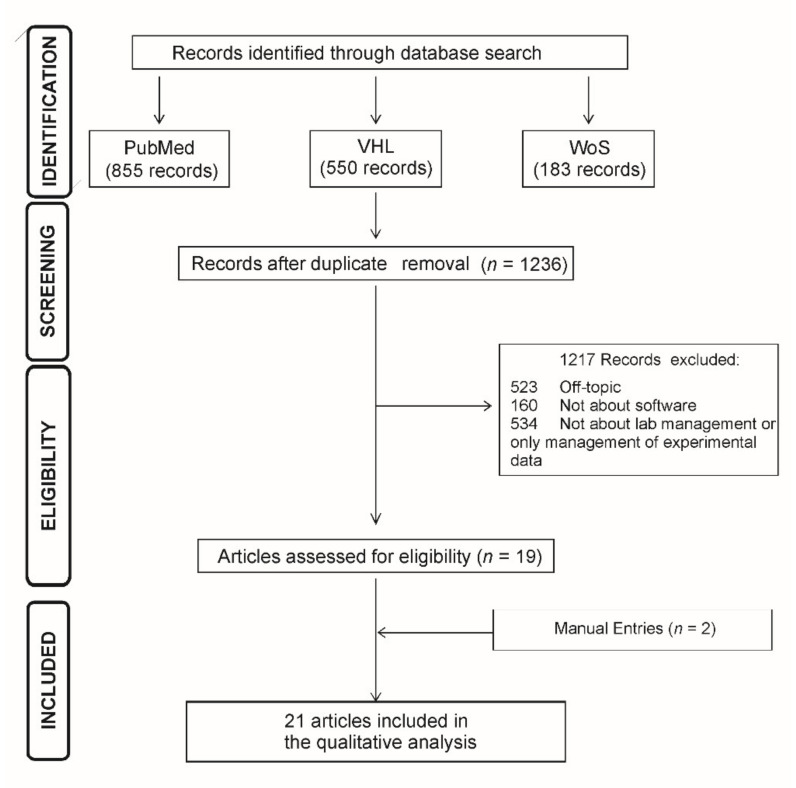
PRISMA flowchart of study screening and selection.

Figure 1 shows the results for the search strategy and screening of databases. The PUBMED database provided 855 entries, while 183 entries were identified in WoS and 550 in the VHL. After combining the 3 results, 352 duplicate articles were identified and excluded. After applying the exclusion criteria, 523 articles were considered off-topic, appearing in searches because of common words and often dealing with clinical/hospital-related issues, but not with experimental medicine. Of the total articles identified, 160 were excluded because they did not deal with software or digital systems, and 534 did not speak about management. From the screening result, 19 articles were selected to compose this Scoping Review, and 2 additional articles were identified manually upon reading the selected references. The twenty-one elected references included studies proposing new software or revisiting already available tools for novel management applications. Some authors also evaluated the impact of changes during and after implementing the systems, either qualitatively or quantitatively.

Table 2 shows a critical appraisal performed for the selected articles at the methodological level and relevance to our broad question. Of the 21 articles selected, 9 evaluated the effectiveness and pointed out the limitations. Another eight did not evaluate but described limitations, and four studies did not evaluate or point out the limitations of the systems used, only describing the implementation or development of the systems in an expository manner. Nevertheless, all articles adequately identified the investigated software, their management purposes, and the environment/professionals served by its functionalities were considered relevant and contributed to some extent to the qualitative discussion on the theme.

Table 3 describes the main characteristics of the twenty-one selected studies related to the present research question. It can be observed that the selection ranged from studies of the earlier days of the use of personal computers in laboratories [15,16] to current cloud computing and mobile applications [25,29]. In addition, some references studied the complexities of the concomitant use of several integrated tools [11,30]. In accordance with the search criteria, the studied environments consisted of academic, health-related laboratories, as well as biorepositories and biobanks. Consistently, the target users were managers and staff common to these laboratories, including technicians, researchers, doctors, and students.

Thirty-three programs/systems were identified in the twenty-one studies, with eight exclusively available for installation on desktop computers and the rest available online, including cloud-based systems, that is, with storage on online servers and availability on demand. Twenty-one of the studied systems were commercially available, charged programs/services, while twelve were free-of-charge for some of their functionalities. Among the non-charged software, two were custom systems designed exclusively for the studied laboratory (Biobank Portal and CCLMS).

Table 4 summarizes the results of the nine studies that assessed the impact of implementing computerized management systems. All of them reported positive results with the use of digital-assisted management. However, problems were identified related to technical constraints (either hardware or software) and limited acceptance of users who resist changing already established procedures, thus impairing the use of some systems to their full potential. Furthermore, the need for staff training and participative management was also recognized to achieve engagement of users to digital-assisted laboratory administration.

Regarding the management subjects issued according to each laboratory, digital systems were employed for several different uses, from purchases and administrative tasks to control of cell collections, inventories in general, as well as data storage and management of animal colonies.

**Figure 2:**
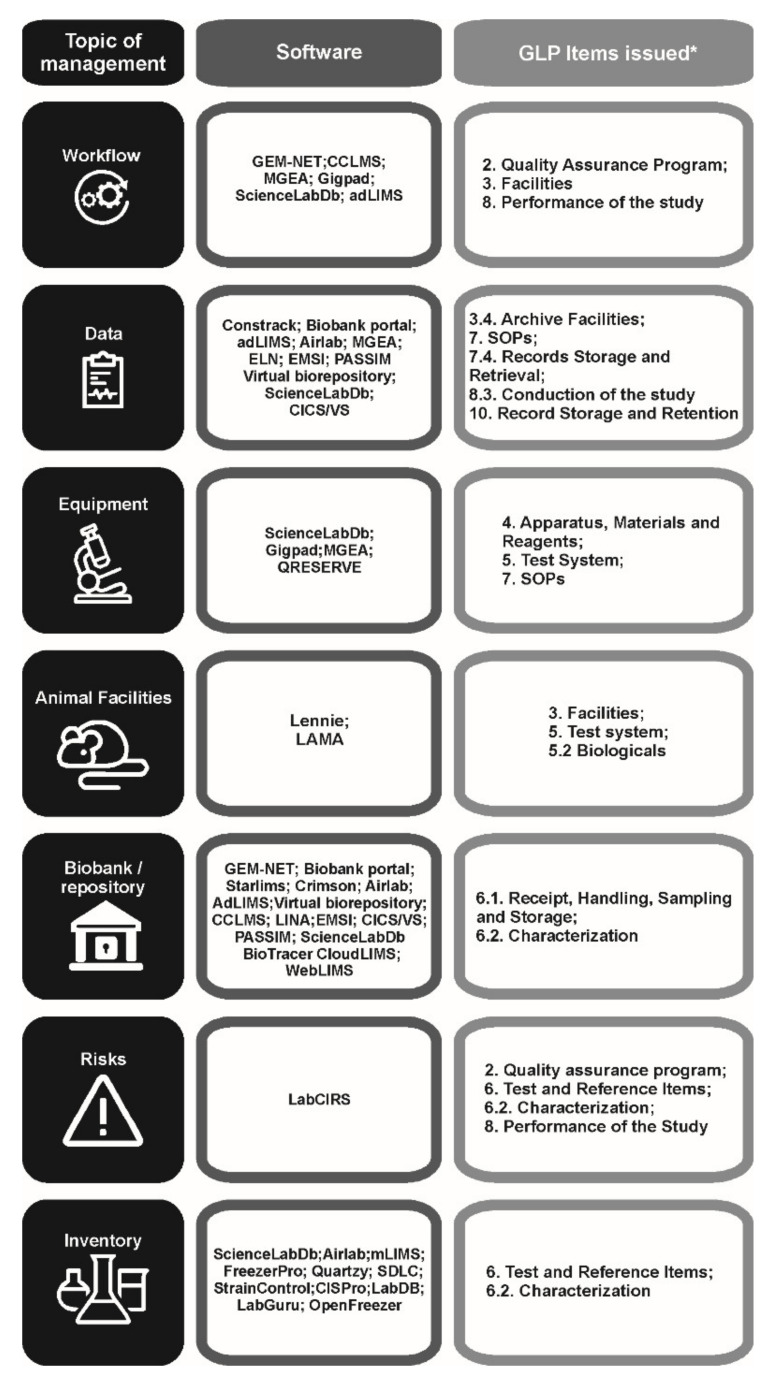
The main applications of the identified software on the different sections and chapters of the OECD GLP Principles [3].

All the thirty-two described software issued one or more topics of management recommended by documents of good laboratory practices [3], including experimental workflow, data storage, integration with laboratory equipment, statistical analysis, comparison of experimental data, animal colonies, biorepositories, inventory, and risks. The integration of work demands of academic health sciences laboratories and items of compliance with the GLP guidelines are identified in the chart presented in Figure 2.

## 4. Discussion

### 4.1. Contributions to Adherence to GLP Principles

While the search strategy from the present review identified several different laboratory management systems, few of the eligible studies provided a focused discussion on this topic. The lack of direct scientific evidence limits the present review to quantitatively assess to what extent digital systems can collectively contribute to accreditation achievement. On the other hand, all the identified software accounted for management issues related to at least one of the GLP principles, and, in some studies, more than one software was used to meet the different demands related to quality systems.

In this sense, the approach proposed by Timoteo et al. [6] could be applied to the present sources of data to chart the main topics of management affected by these programs and systems related to good practice guidelines. The chart presented in Figure 2 shows how the types of management supported by the software in academic laboratories are related to several items from Section II of the OECD GLP Principles [3]. Such relationship is revealed by an emphasis on the responsibilities of staff and facilities management, work planning, availability of standard operational procedures (SOPs) that cover all study activities, procedure analysis, use and maintenance of equipment, as well as the application of standards for receiving test samples, its chain of custody and logistics, control of inventory, and the traceability of reagents and validation of methods.

For a better understanding of the functions of these systems, a brief presentation of them will be made, with an emphasis on meeting the computerized systems to the GLP principles listed in Figure 2.

#### 4.1.1. Workflow

The GLP principles require precise definitions of the different steps during the performance of the study, as described in item #8 of the OECD document [3], including the responsibilities of the personnel involved, the facilities and status of equipment employed (item #3), among other factors. Furthermore, quality assurance (item #2) requires identifying and monitoring critical steps, checkpoints, and possible sources of errors. Among the different systems identified in the present review, some described digital tools dedicated to managing such workflow of study performance in a systematized fashion.

In the late 1990s, Goodman and colleagues [16] presented Labflow, a software dedicated to genetics and mapping studies. Workflow management was not recognized as a study topic at that time and, while LIMS already existed, there was no commercial LIMS product that supported workflow management in a specific sense. In this scenario, LabFlow appeared among the first digital solutions, with a workflow model in which objects flow different laboratory tasks (such as DNA extraction, selection of clones, sequence analysis) under programmatic control. An essential point of this software was already allowing the programmer to customize their workflows to different laboratory needs.

Anderson et al. [19] described, in 2007, the implementation of the Microarray Gene Expression Analysis (MGEA), a software package developed by Rosetta Biosoftware (a subsidiary of Merck Inc.), that helped to integrate workflow information related to experimental design, data collection, and bioinformatic analysis of genomic results. Despite the high costs of the license and its renewals, the authors expected that implementing a commercially available service would bring advantages such as security in terms of support for operation and uniformity between different research centers, thus facilitating communication between employees. However, their qualitative analysis observed that the system was not used to its full potential, and its acceptance by staff would demand ongoing training and even an evolution of academic curricula towards the use of bioinformatics tools.

In 2019, Gaffney et al. [11] described the design and implementation of GEM-NET, a software that allowed members of the C-GEM (Center for Genetically Encoded Materials, USA) to integrate research efforts connecting six laboratories spread across three university campuses. GEM-NET was designed to support science and communication by integrating task management, scheduling, data sharing, and internal communications. A set of more than 20 tools was organized, including two applications customized for the Institution’s specific needs of workflow management. The tools are highly interconnected, but the set can be divided into access control, data storage, data navigation, project monitoring, teamwork, internal communication, and public engagement. The authors conclude that GEM-NET provides a high level of security and reliability in workflow management.

#### 4.1.2. Data Management

In different items of the GLP principles, a need is described for the secure storage, filing, and retrieval of research data (item #7.4), including study plans, raw data, final reports, test system samples, and specimens (item #8.3), and their related archiving facilities (item #3.4). Furthermore, item #7 (standard operating procedures) requires the preparation and observance of documents that guarantee the quality and integrity of the data generated by the studies. Sub-item #7.4, for example, describes that in the case of computerized systems, validation, operation, maintenance, security, change control, and the backup system must be observed.

Within the selected studies, we found the report of computerized systems to manage data from various laboratory environments and how they were made available to the research groups. In the early 1980s, Delorme and Cournoyer [15], in a microbiology laboratory of a University Hospital, tested the CCIS/VS (Customer Information Control System/Virtual Storage), consisting of customer data repository, using a central computer shared with medical records databases, admission offices, patient accounting, and other medical-administrative services. The system also served as a virtual storage system, including data from microbiological samples. It performed activities such as report printing, data quality control, epidemiological assistance, germ identification, teaching, and research in the different subspecialties of microbiology. The authors carried out a qualitative and quantitative assessment identifying an improvement of workflow without increasing personnel, together with a reduction in the time for the production of reports, system downtime, and other parameters.

Viksna et al. [20] focused on collecting, storing, and retrieving data on research participants and biomedical samples through electronic management. For this, they proposed the PASSIM (Patient and Sample System for Information Management), a web-based customizable system that could be used for sending, managing, and retrieving samples and data from the research subject, ensuring the confidentiality of the records. This tool was instrumental in managing information in clinical research studies involving human beings and replaced the more expensive LIMS, which requires investments of time, effort, and resources that were not always available.

Electronic laboratory notebooks (ELN) are programs designed to replace traditional research notebooks. These electronic tools may register protocols, field/lab observations, notes, and other data inserted through a computer or mobile device, offering several advantages over paper notebooks [19]. Machina and Wild [22] investigated the importance of ELNs when integrated with other computer tools, such as laboratory information management systems, analytical instrumentation, data management systems, and scientific data. They observed that the type of laboratory (analytical, synthesis, clinical, research) was a primary source of differences when trying to integrate ELN with the available tools. Therefore, based on the observation that there was no well-established path for the effective integration of these tools, the authors decided to review and evaluate some of the adopted approaches.

Calabria et al. [24], in 2015, introduced adLIMS, a software for managing biological samples (primarily DNA) and metadata for patient samples and experimental procedures. The authors described how it was possible to produce this system by customizing a previous open-source software, ADempiere ERP. First, they collected the requirements of the end-users, verifying the desired functionalities of the system and Graphical User Interface (GUI), and then evaluated the available tools that met the desired requirements, ranging from pure LIMS to content management and corporate information systems. The authors report that the system supported critical issues of sample tracking, data standardization, and automation related to NGS (next-generation sequencing).

By 2021, Cooper et al. [30] reported using integrated systems that ensure the sharing of essential data for current research. The authors followed the 15 years of development and implementation of the LabDB system, initially projected to manage structural biology experiments, which could be improved into a sophisticated system that integrates a range of experimental biochemical, biophysical, and crystallographic data. The LabDB central software module handles data from the management of laboratory personnel, chemical stocks, and storage locations. It is currently used by the American/Canadian consortium CSGID (Center for Structural Genomics of Infectious Diseases) and several prominent research centers. The authors identified the difficulties and resistance of some researchers in adopting these systems as the main limitation, often due to the necessary effort to import data from electronic notebooks or laboratory spreadsheets, with which most researchers are already familiar. Nevertheless, the authors consider that this effort is worth it since these older approaches do not remove or even track inconsistencies and do not adapt well to the requirements of modern research.

It is essential to notice that, for accreditation purposes, hosted services (cloud archiving, backup, or processes) require written agreements describing the responsibilities of the informatics services. Test facility management must be aware of potential risks on data integrity resulting from third-party storage.

#### 4.1.3. Equipment

Adherence to the GLP principles speaks to the adequate management of research equipment (OECD item #4), including their adequate calibration, maintenance, scheduling, and responsible staff in the test facility. Several commercially available systems, such as QRESERVE, cited by Perkel [9], are entirely dedicated to these functions, with integrated reservation calendars, administration of equipment status and availability, a repository of maintenance documentation, and a registry of use time. Other all-purpose management systems such as Labguru have most of these functions on a specific equipment module. That was also the case of the freely available (for individual researchers) Quartzy until 2016, as reported by Timóteo et al. [6]. This study described how the implementation of the software optimized the shared use of equipment on a multiuser clinical research unit and the advantages of allowing equipment scheduling, check-in, and check-out remotely, even using mobile phones.

#### 4.1.4. Animal Facilities

Several procedures related to pre-clinical studies conducted with animals are issued in the GLP principles, mostly in item #5 of the OECD document (test system) and subsection #5.2 (Biologicals). These include a proper registry of housing, handling, and care of animal test systems to ensure the quality of the data. Additionally, records of source, date of arrival, and arrival condition of test systems should be maintained. Two selected studies described the use of vivarium monitoring software to ensure the remote control of stocking, accommodation, handling and care of animals, identification of colonies, and inventory of supplies.

Milisavljevic et al. [8] described, in 2010, the Laboratory Animal Management Assistant (LAMA), a software modified from the LIMS proposal to optimize small animal research management. It was initially developed to manage hundreds of new mouse strains generated by an extensive functional genomics program in Canada. The authors realized that they needed greater availability of suitable, easy-to-use systems and software interfaces. LAMA was implemented for a broad community of users, allowing individual research labs to track their colonies in a larger facility, independently. This open-access software is still available to the research community.

Allwood et al. [23] described, in 2015, how smartphones could help researchers in the remote management of animal colonies. The authors proposed Lennie, an app that introduced a new method for managing small to medium-sized animal colonies, allowing users to remotely access the facilities, and create and edit several functions virtually from anywhere. Its use contributes to the optimization of workflow and planning of experiments, offering a user-friendly experience. Possible updates to the functionalities were also suggested, such as camera integration with the calendar, permission for data sharing, and permanent storage.

#### 4.1.5. Biobank/Repository

In order to comply with the GLP standards, samples that arrive at a laboratory must have records that include the characterization and reference, date of receipt, expiration date, quantities, and storage data, following item #6.1 (receiving, handling, sampling, and storage). This issue is of utmost importance for managing biobanks and biorepositories, creating a need for specific software for successful management.

Boutin et al. [25] carried out a study on a complex system of various software that contributed to the management of a Biobank. The core object of management was an extensive repository of samples and data available to researchers. The platform requires robust software and hardware, as they work with large amounts of data stored and transferred to research groups. In the study, the authors described each of the five custom and commercially available information systems integrated into the existing clinical and research systems, and discuss safety, efficiency, and challenges inherent in the construction and maintenance of this infrastructure. Constrack was used to manage patient data. The Enterprise Master Specimen Index (EMSI) is a sample indexing system, STARLIMS manages inventory, GIGPAD manages data and integrates equipment, and the Biobank Portal is the customized application that connects all the systems.

Manca et al. [28] assessed the structure of a central laboratory of the Antibacterial Resistance Leadership Group (ARLG) in the USA. This group leads the evaluation, development, and implementation of laboratory-based research and supports standard or specialized laboratory services. The laboratory included both a physical and a virtual biorepository. They developed digital procedures for reviewing and approving strain requests, providing guidance during the selection process, and monitoring the transfer of strains from the distribution laboratories to the requesting investigators.

Paul et al. [29] also describe a Biobank management system, with great emphasis on data storage in clouds. The authors evaluated that biobanks have become an essential resource for health research and drug discovery. However, collecting and managing large volumes of data (bio-specimens and associated clinical data) requires biobanks to use more advanced data management solutions. Paul and Chatterjee [27] point out that in the current COVID-19 pandemic scenario, that requires global and quick actions, virtual biobanks present a crucial role in several different fronts, from diagnosis to research. Without the need to physically use biological samples, these banks may allow sharing medical data and networks for better cooperation between biobanks at the national and international levels.

Recently, Dennert, Friedrich, and Kumar [1] explained the various implications of the inventory management of biological samples from various research areas, employing different cryopreservation methods. Such management must ensure the availability of items, easy tracking, and the optimization of shared space among the various research groups. For this, the authors presented the various stages of developing an inventory data model using the Microsoft Access database, after several phases that included training, planning, implementation, and maintenance, as well as the establishment of manuals and protocols for standardized data entry. Using the software development lifecycle (SDLC), the authors attained a database construction model. This model requires frequent communication with users to provide transparency and quality improvement.

#### 4.1.6. Risk Management

Identifying incidents and risk assessment is an essential part of the GLP standards that requires an adequate work plan and a quality assurance program (OECD document item #2). Item #8.3 of the GLP states that all data changes during the conduction of a study must always be registered and responsible for the change to ensure traceability, enabling a complete audit trail to show all changes without masking the original data.

The work of Dirnagl et al. [27] discusses how error management is fundamental to comply with international standards while studying the implementation of the LabCIRS (Laboratory Critical Incident Reporting System), a simple, accessible, and open-source critical incident reporting system for pre-clinical and basic academic research groups. The software was implemented by establishing an electronic quality management system, which allowed accessibility through any laboratory computer, enabling incident reports that included photo uploads and automatic alerts for new reports and archiving.

#### 4.1.7. Inventory

Item #6.2 of the GLP principles clearly states that all material from a study must be adequately identified, including the batch number, purity, composition, concentrations, or other characteristics, to define each item or reference item properly. It also indicates the need to keep the receipt and expiration dates, quantities received/used in the studies, and storage instructions for the stock of materials. In this review, several articles emphasized this need to monitor inventories with the help of computerized systems.

Nayler and Stamm [17], in 1999, described a laboratory management software, ScienceLab Database (SLD), which offered a management platform for molecular biology research laboratories. The program primarily manages the stock of biological samples, including plasmids, antibodies, cell lines, and protocols, and included an ordering and grants management system. The authors considered that this system met the specific needs of a small to medium-sized research laboratory, helping to organize inventories of valuable reagents, storing, and maintaining information about these items, and simplifying orders and processes.

By 2016, Catena et al. [26] developed the AirLab, a cloud-based tool with web and mobile interfaces, to organize antibody repositories and their multiple conjugates. Due to the large number of data generated by these collections, the authors recognized the need for dedicated software. The work demonstrated that Airlab simplifies the purchase, organization, and storage of antibodies, creating a panel to record results and share antibody validation data.

Yousef et al. [21] described the LINA (Laboratory Inventory Network Application) as a set of Windows-based inventory management software configured to work on a computer network with multiple users. Designed for small molecular biology laboratories, it uses Access databases to assign a new identifier to each new reagent, providing a library that helps with research and comparing DNA sequences. It later faced several features, such as expanding the types of tables available, compatibility with other operating systems, barcoding, and improvement of security issues. According to the authors, the resources provided by LINA are comparable to those available in commercial databases, with the advantage of providing a free database maintenance application for academic laboratories.

In an opinion article published in Nature’s section “Toolbox”, Perkel [9] describes several low-cost computerized electronic inventory systems as a means to overcome tortuous searches, old notebooks, out-of-date spreadsheets, and “frost-encrusted freezer boxes” to identify laboratory samples and resources. Besides programs discussed by other authors in this review, such as LINA and Quartzy, the article cites other systems such as OpenFreezer, a free web-based system to register sample data such as location, origin, and biological properties, the cloud-based StrainControl (DNA Globe, Sweden), a software free for individual researchers that provides support for managing different lab-organism strains, molecules, and chemicals, the mLIMS, developed by BioInfoRx (Madison, WI, USA), designed to track rodent colonies, LabGuru (BioData, Cambridge, MA, USA), a widely known paid cloud-based all-in-one Electronic Notebook, and CISPro (BioVia, Waltham, MA, USA), described as a functional Institute-wide tracking system for shared resources. Despite differences in accessibility and several resources, all of these systems share similar search engines linked to customizable databases.

Timoteo et al. [6] evaluated, by 2020, the impact of implementing a multi-module, free-of-charge online management system (Quartzy, Quartzy Inc., Santa Clara, CA, USA) in the workflow of a Brazilian academic clinical research laboratory on the perception of users. Until 2016, the software modules could assist in various aspects and demands of the laboratory, including user communications, multiuser equipment management, material inventory, research documents, and tracking of supply orders. Unfortunately, Quartzy was recently updated to a simpler version, consisting only of an inventory and purchase tracking system that connects researchers to hundreds of life sciences brands and suppliers.

### 4.2. Evaluating Impacts and Limitations

Effectiveness is a fundamental point to be considered in the potential role of software for laboratory management. However, most of the eligible studies identified in our search did not investigate the reported systems’ impact either through qualitative or quantitative assessments. Moreover, despite the performance of evaluations, few studies identified or discussed the limitations and drawbacks of the studied information systems. The studies with evaluations reported, among several aspects, improvement of the organization, workflow, traceability, reliability, acceptability, and good use of the software. Decreased process errors were reported that were made manually, thereby gaining productivity and reducing work. In some specific cases, they positively evaluated the control of frozen cells, generating efficiency and better results in partner laboratories. On the other hand, regarding limitations, older articles (before 2000) identified problems that were more related to system performance, which was sometimes slow and needed adjustments at a time when information technology was still incipient. The limitations from the most current systems are more related to a selective satisfaction and acceptance of software tools, specific according to the function and objective of each group and, in some cases, the resistance by researchers and staff to abandon old ways and migrate to digital tools, which were not used to their full potential within the laboratory.

To adequately assess the impact of these electronic management systems, different methodological approaches are available, such as pre/post-tests evaluating quantitative indicators of performance and provision of services. However, as Timoteo et al. [6] discussed, the complex nature of the provided services of multiuser, academic research facilities may impair the obtention of feedback through quantitative indicators. In this sense, the perception and attitudes of staff towards the management system may contribute to understanding its impact on the workflow and the search for quality at academic clinical research laboratories, as well as provide data for the development or improvement of actions and strategies toward quality and compliance [31,32,33]. In this sense, validated tools may provide a means to standardize the evaluation of laboratory management software, allowing comparisons on the effectiveness and adequacy of these systems in different applications. Two studies [18,21] proposed the use of an important tool to investigate the effectiveness and efficiency of the software, the system usability scale (SUS). This tool, developed by John Brooke at Redhatch Consulting (UK), consists of a simple, ten-item attitude questionnaire using a Likert scale to provide a global view of subjective assessments of usability, which was validated as providing reliable results even with small samples/study groups, which was the case of most identified studies in this review. Therefore, it may represent a potential tool (although underestimated until the present moment) for further studies on implementing laboratory management systems.

Different studies point out that staff training is one of the most important factors of success of the implementation of these systems and a key part in acceptance and adapting to a new management model. Dirnagl et al. [27] evaluated the impact on staff attitudes toward incident reporting after one year of implementation, observing that training led to greater adherence to the goal of complying with international quality standards and mature culture of error management. Timóteo et al. [6] performed a qualitative evaluation of the staff perception on software implementation, where most users stated that constant training and leadership were pivotal for the successful use of the software. On the other hand, Anderson et al. [19] reported that limited access to training was a barrier to software use during the implementation of MGEA, and that the lack of ongoing training might have contributed to a progressive de-emphasizing of the system use among the laboratory staff. These data point to the need of careful planning by the PIs to ensure continuous and inclusive training on the implementation program of management systems.

### 4.3. Software Availability

Regarding availability and accessibility, until 2010, most of the identified programs had to be downloaded/installed to specific laboratory computers [19,30], but were sometimes able to integrate local area networks (LANs), as described by Delorme and Cournoyer in 1980 [15]. In the past decade, technology has advanced to online software, expanding even to applications (apps) on mobile phones, reflecting the current expectations of users and consumers. With app technology permeating all fields of our daily lives, it would be natural for this technological paradigm to reach laboratory and research technologies. Indeed, a big leap was identified towards the proper integration between lab management systems and the new mobile universe. Real-time communication makes it possible, for example, that inventory checks, equipment scheduling, and data verification of an animal colony be performed while in transit. Multicenter studies can share data in real-time, as recently observed in the fast development studies of vaccines against SARS-CoV-2 since 2020, relying heavily on technological development and efficient data management [34].

Begg et al. [35] discussed how computer systems are of particular importance in the process of GLP certification in low- and middle-income countries, even though their role is not always emphasized on accreditation systems around the world. This review identified that the knowledge on laboratory management software is mainly originated, as expected, from developed, high-income countries, with advanced information technology industries and significant investment in technology and support for universities and study centers (USA, Germany, Canada, United Kingdom, Switzerland). In a critical view, it may indicate an economic bias in the technological development on the theme, as developing countries maintain a role as consumers of technology and not as producers and developers, reflecting little investment in this (and other) technological areas.

The costs of implementing computerized systems may represent one of the main challenges for public Academic Health Centers since these Institutions, in general, face tight budgets to support several laboratories, researchers, and research lines. Such limitations are expected to be potentialized when considering low- to middle-income countries, which could benefit from low-cost or cost-free initiatives.

In general, the development and maintenance of information systems are made possible by providing subscription services to ensure the tool’s sustainability. The present review identified some systems that addressed a full spectrum of fundamental issues in the management of academic laboratories, such as inventory control and organization and equipment scheduling, on a free-of-charge basis, as it incorporated catalogs from various sponsors (reagent suppliers) and suggests these products when orders are placed [9]. However, such a business model probably did not match the maintenance costs of the platform, as Quartzy has shut down all functions not related to inventory/purchases by 2016, and recently included a fee for Institutional users. It is also possible that users from outside the USA and Europe could not use the vendor-related functionalities, as customer services and representatives in regions such as South America would not connect directly to the system [6]. On the other hand, LINA is an example of a system that could remain free-of-charge, even though limited to the needs of small molecular biology laboratories [21], with much simpler functionalities compared to well-known commercial applications such as Labguru. Other services, such as QReserve, have both free and paid versions with increased functionalities, allowing low-budget academic laboratories to use some free resources, such as equipment reservation and management, through a more straightforward interface.

A usual profile among entirely free software originates from in-house academic software, such as Biobank Portal and CCLMS, customized for the personal use of the developer group, usually without widespread use in other institutions. Even though they may present advantages on issuing specific demands of developers, the lack of a profound, systematic evaluation of performance on most selected studies does not allow to infer whether these are more or less effective than commercial software. In this sense, Boutin et al. [25] report that the laboratory IT framework may face challenges common to industry settings, where cost-overrun is prevented by planning the cost-effectiveness of purchasing commercially available vs. designing in-house custom applications. An interesting way to achieve broader applicability for such software is to use open-source codes, such as Boutin et al. [25], paving the way for other programmers to adapt the tool to different laboratory specificities. It is important to notice that investments from government bodies worldwide could also contribute to the development of freely available tools as part of public policies focused on increasing overall quality and adherence to good practices in health sciences research. In this sense, the encouragement of startups involving interdisciplinary initiatives can turn universities and academic centers into important stakeholders in covering technological gaps in low- or middle-income countries [36].

### 4.4. Review Limitations

The present Scoping Review has limitations mainly related to the impossibility of exhausting the literature on laboratory software, reflected in the choice of not including programs that dealt only with the transmission and handling of analysis results and laboratory data, such as pure LIMS or analytical bioinformatics software. Despite their fundamental role, these types of software have already been widely discussed [37,38,39,40], and most of these systems were not designed to support the management of staff and shared resources, for example. Additionally, the scientific literature probably does not reflect the abundance of available software since developers and the scientific community usually treat them as a commercial tool rather than a research topic. Nevertheless, regardless of such limitations, the present review was able to map a framework that points to the great applicability of these systems in the search for quality and good practices in academic experimental medicine laboratories, where restrictions regarding the availability of resources and staff and limited management experience are common restrictions. Therefore, the gaps identified here can serve as an indication for new studies that seek to assess, quantitatively or qualitatively, the impact of implementing these tools on the best practices at academic health Institutions.

## 5. Conclusions

The present literature review mapped several studies in the last four decades, proposing and evaluating the impact of digital tools in the management of health sciences research laboratories to several different applications, ranging from administrative workflow management and data traceability to virtual biobanking. These functions have the potential to contribute to the adherence to different GLP principles. However, the evidence for their effectiveness is still limited and requires further investigative efforts.

## Figures and Tables

**Table 1:** Search keys applied to the three consulted databases.

Database	Search Key
PUBMED	(laborator*[tiab] OR Laboratories[mh]) AND (management[tiab] OR “Organization and Administration”[mh] OR “Information Management”[mh]) AND (software[tiab] OR computer*[tiab] OR virtual[tiab] OR Software[mh] OR “Mobile Applications”[mh]) AND (academic OR Universities[mh] OR research[tiab] OR research[mh] OR “Biomedical Research”[mh] OR “Translational Medical Research”[mh]) AND (health OR clinic*)
Web of Science	TOPIC: ((laboratory) AND (management OR “Organization and Administration” OR “Information Management”) AND (software OR computer OR virtual OR “Mobile Applications”) AND (academic OR University OR research OR “Biomedical Research” OR “Translational Medical Research”) AND (health OR clinic)). Time stipulated: all years. Indices: SCI-EXPANDED, SSCI, A&HCI, CPCI-S, CPCI-SSH, ESCI.
Virtual Health Library	(laboratory) AND (management OR organization) AND (software OR computer OR virtual OR “Mobile Applications”) AND (academic OR University OR research) AND (health OR clinic)

**Table 2:** Critical appraisal of the sources of evidence.

	Adequacy to the Research Question		Methodological Soundness		
Reference	Description of Software Limitations	Description of Functions and Users/Environment	Evaluation of Applicability, or Acceptance	Adequate Identification and Traceability	Final Score
Delorme and Cournoyer [15]	1	1	1	1	4
Godmann et al. [16]	1	1	0	1	3
Nayler and Stamm [17]	1	1	0	1	3
Selznick et al. [18]	1	1	1	1	4
Anderson et al. [19]	1	1	1	1	4
Viksna et al. [20]	0	1	0	1	2
Milisavljevic et al. [8]	1	1	0	1	3
Yousef et al. [21]	1	1	1	1	4
Machina and Wild [22]	1	1	0	1	3
Allwood et al. [23]	1	1	0	1	3
Calabria et al. [24]	1	1	1	1	4
Perkel [9]	0	1	0	1	2
Boutin et al. [25]	0	1	0	1	2
Catena et al. [26]	1	1	0	1	3
Dirnagl et al. [27]	1	1	1	1	4
Manca et al. [28]	1	1	1	1	4
Paul et al. [29]	0	1	0	1	2
Gaffney et al. [11]	1	1	0	1	3
Dennert Friedrich and Kumar [1]	1	1	1	1	4
Timoteo et al. [6]	1	1	1	1	4
Cooper et al. [30]	1	1	0	1	3

To quantify the criteria, “1” means present, and “0” means absent.

**Table 3:** Main characteristics of the selected studies.

Reference	Country	Software	Availability	Managed Activity	Environment	Target Users	Costs
Delorme and Cournoyer [15]	UK	Customer Information Control System/Virtual Storage (CICS/VS)	Desktop	Tax and administrative tasks, quality control of data and techniques, epidemiological assistance, and teaching and research in the different subspecialties of microbiology.	Microbiology laboratory at a university hospital	Medical Doctors, researchers, and students	Charged
Godmann et al. [16]	USA	LabFlow	Desktop	Workflow in large-scale biology research laboratories.	Research Laboratory	Researchers and laboratory users	Free
Nayler and Stamm [17]	Germany	ScienceLab Database (SLD)	Desktop	Stock of reagents and biological samples, protocols, library, vendor information.	Molecular biology laboratory	Laboratory professionals	Charged
Selznick et al. [18]	USA	Cell Culture Laboratory Management System (CCLMS)	Desktop	Cell culture laboratory management: modules for registering cell counts, frozen cell records, user records, and culture vessel specifications.	Cell culture laboratory	Researchers and users of cell culture laboratories	Custom prototype
Anderson et al. [20]	USA	MGEA	Desktop	Experimental workflow, integration with laboratory equipment, storage, and statistical analysis of experimental data.	Genetic research laboratory	Researchers, laboratory professionals, biostatistics, students.	Charged
Viksna et al. [20]	UK	Patient and Sample System for Information Management (PASSIM)	Desktop/ online	Study participants, samples, and results.	Biorepository and biomedical research labs	Researchers and students	Free and open source
Milisavljevic et al. [8]	Canada	Laboratory Animal Management Assistant (LAMA)	Online	Management of mouse colonies.	Biotery.	Researchers	Free
Allwood et al. [23]	Canada	Lennie	Smartphone	Maintenance and management of animal colonies.	Vivarium.	Researchers	Free
Yousef et al. [21]	USA	LINA	Desktop	Track collections of biologically relevant materials.	Molecular biology academic laboratories.	Medical Doctors, researchers and students	Free
Machina and Wild [22]	USA	Electronic Laboratory Notebook (ELN)	Desktop	Automation of lab tests; register of equipment-related data (use, and calibration). Laboratory inventories.	General laboratories.	Researchers and laboratory users	Charged
Calabria et al. [24]	USA	AdLIMS	online	Biological samples; metadata from patient samples; experimental procedures, workflow, and data for DNA samples.	Genetic sequencing laboratories.	Researchers and users of cell culture laboratories	Charged
Perkel [9]	USA	Quartzy; LabGuru; LINA; StrainControl; CISPro; mLIMS; OpenFreezer	Online/ smartphone	Sample tracking and inventory.	Research laboratories and Academic Institutions	All levels of laboratory staff	Free and charged tools
Boutin et al. [25]	USA	STARLIMS; GIGPAD; Crimson; Constrack; EMSI; Biobank portal	Online/ smartphone	Genomic data transfer, sequencing, genotyping, sample inventory, workflow, DNA and RNA sample processing and tracking; patient data.	Research laboratories, biobanks, collection clinics, hospitals	Coordinators, research subjects, researchers, IT staff.	A combination of custom/ free/ charged tools
Catena et al. [26]	Switzerland	AirLab	Online/ smartphone	Reagent and sample inventory; database of antibodies.	Research laboratories, mainly molecular.	Researchers and laboratory students	Free
Dirnagl et al. [27]	Germany	LabCIRS (Laboratory critical incident report)	Online	Risk/error management.	Research Laboratories	Research groups, laboratories, and institutions	Free
Manca et al. [28]	USA	Laboratory Center (LC)	Online	Virtual biorepository.	Antibacterial Research Laboratory; biorepository	Researchers	Charged
Paul et al. [29]	USA	BlazeLIMS; FreezerPro; WebLIMS; BioTracer	Cloud	Biobanking.	Biobanks	Doctors and researchers	Charged
Gaffney et al. [11]	USA	GEM-NET	Online	Access control, data and protocol storage, project monitoring, teamwork, internal communication, engagement, and biorepositories.	Academic Research Laboratories—Biorepository of specimens	Researchers and laboratory students	A combination of free/charged tools
Timoteo et al. [6]	Brazil	Quartzy	Online	Staff and workflow management of an academic research lab including documentation, equipment, inventory, and communication.	Academic Research Laboratories	Researchers, and laboratory users.	Free/charged versions
Dennert, Friedrich and Kumar [1]	USA	Database—SDLC System	Online; LAN.	Inventory Management System	Research laboratories; Biorepository of specimens	Researchers and laboratory staff	Charged
Cooper et al. [30]	USA	LabDB	Online; LAN.	Manages experimental data and organizes personnel and inventory.	Research laboratories.	Researchers	Charged

**Table 4:** Results of the studies that assessed the impact of implementing computerized management systems.

System/Software	Reference	Objective	Test Groups	Method	Results
Customer Information Control System/Virtual Storage (CICS/VS)	Delorme and Cournoyer [15]	Qualitative and quantitative evaluation of system limitations and impact on workflow and man/hour relationships	Software developers and users at the Hospital Lab (N.D.)	Qualitative evaluation of the development of integrated modules; during field testing, the workflow was accessed by the evaluation of patient entry forms, the results of sample and data processing, and the final reports.	− Technical limitations were identified in the software and hardware; changes on the systems solved software-related issues. − More accurate patient and sample reports; control over the destination of the requested tests; easier control of billing; faster delivery and retrieval of results.
CCLMS	Selznick et al. [18]	Test system improvement on organization and control of collections.	Cell culture specialists from 2 labs (n = 6)	Qualitative and quantitative evaluation of usability through the system usability scale (SUS) and field notes.	− CCLMS improved the laboratory’s organization set, increased efficiency and reliability.
MGEA	Anderson et al. [19]	Assess the impact on experimental workflow for gene expression analysis.	Researchers (n = 7)	A qualitative longitudinal study. Immersion in the work environment. Interviews, observations, and field notes were coded and analyzed.	− The system performed as a measurement tool rather than the “total laboratory analysis solution” desired initially. − The acceptance of software tools was specific to their function and objectives. − The tool was not used to its full potential.
LINA	Yousef et al. [21]	Effectiveness and acceptance of an inventory system for management of oligonucleotides, strains, and cell lines	Lab staff (n = 10)	Qualitative analysis of the implementation process; quantitative evaluation of usability through the SUS.	− The LINA project achieved its original objectives, as the system obtained an adequate mean SUS score of 86.25.
AdLIMS	Calabria et al. [24]	Evaluate effectiveness on sample tracking in genomic studies.	Developers and potential users/clients (N.D.)	Analysis of requirements and expectations of functionalities from users/clients in terms of functionalities; qualitative analysis of the development process.	− Improved workflow by reducing the time spent on repetitive tasks through interfaces with smartphones and tablets. − Reduced manual errors, standardizing pharmacovigilance monitoring of gene therapy patients. − Compatible with regulatory requirements.
LabCIRS	Dirnagl et al. [27]	Assess the acceptability, usability of a software of risk assessment for traceability of reported cases.	Lab staff (n = 31)	Statistical and qualitative analysis of the data before and after the implementation of the tool. Online questionnaire with two questions on software usability	− Increased responsibility and maturity to deal with and prevent errors. − Differences in the frequency of digital and paper reporting. − Increased quality, safety, and communication. − Improvement of prevention policies.
LC Virtual Biorepository of the Antibacterial Resistance Leadership Group (ARLG)	Manca et al. [28]	Assess the impact of the implementation of a virtual repository on the management of data and biological collections.	Customers from research labs and diagnostic companies (N.D.)	Qualitative evaluation of the efficiency of the primer bank sequences. Quantitative retrospective assessment of impacts on services provided.	− The software provided sound technical and scientific support to diagnostic companies and platforms. − More than 200 samples/year were provided for research laboratories and diagnostic companies.
Quartzy	Timoteo et al. [6]	Assess the impact of the implementation in the workflow and the perception of users at an academic laboratory.	Lab staff (n = 30)	Qualitative analysis of the team’s attitude towards implementation, including a structured questionnaire (and focus group assessments). Management performance indicators were also compared before and after implementation.	− There was a perception of improvement in the workflow in relation to the organization, data logging, traceability, distribution, and overall workflow. − Constant training and a management plan are essential if the potential use of supporting software is exploited to the full.
SDLC	Dennert, Friedrich, and Kumar [1]	Evaluate the development steps of a database of biological sample inventories	Researchers from different fields of medicine (N.D.)	Immersion in the work environment: The cycles of all resources have been developed and tested. User training and interviews were conducted to assess the applicability and identify user’s needs.	− The efficiency, traceability, and cost savings led to significant improvements in the workflow and consolidated inventories, reducing storage needs.

N.D.: non-determined number of participants.

## Data Availability

The data presented in this study are openly available in the Open Science Framework (OSF) database, at doi:10.17605/OSF.IO/KPC3Q.

## Supplementary Materials

The following are available online at https://www.mdpi.com/article/10.3390/healthcare9060739/s1, Table S1: Preferred Reporting Items for Systematic reviews and Meta-Analyses extension for Scoping Reviews (PRISMA-ScR) Checklist.
